# P01.25. Classification of L6 muscle spindle afferents in the anesthesized cat

**DOI:** 10.1186/1472-6882-12-S1-P25

**Published:** 2012-06-12

**Authors:** W Reed, J Pickar

**Affiliations:** 1 Palmer College of Chiropractic, Davenport, USA

## Purpose

Patients with low back pain demonstrate proprioceptive difficulties including spinal repositioning errors and impaired lumbosacral proprioceptive acuity. Few data are available regarding proprioceptive properties of muscle spindles in the low back. Muscle spindle afferents can originate from 2 types of receptive endings which terminate and respond to mechanical changes in 3 types of intrafusal fibers. Receptive endings may be primary or secondary. They terminate on dynamic bag (b_1_) static bag (b_2_) and/or chain (c) intrafusal fibers. We sought to classify lumbar paraspinal muscle spindle afferents based upon their receptive endings and intrafusal terminations. Classification was based on their responses to ramp and hold vertebral movement before and after intrafusal activation using succinylcholine (SCH, 100-300ug/kg.ia). Afferents terminating in primary endings are especially responsive to the dynamic ramp stimulus. During intrafusal activation, afferents terminating on b_1_ fibers further increase their discharge to the dynamic ramp whereas those terminating on b_2_ fibers increase their static resting discharge.

## Methods

Electrophysiological recordings from spindle afferents (n=195) were obtained from L_6_ dorsal root filaments with receptive fields in the L_6_ longissimus and multifidus muscles in an anesthetized cat preparation. Controlled vertebral actuations that stretched the paraspinal muscles were applied to the L_6_ spinous process in a dorsal-ventralward direction [1.5mm (n=120), 1.6mm (n=21), or 1.7mm (n=54) using a feedback motor system. Instantaneous discharge frequency was averaged and compared over three ramp cycles pre- and post-SCH injection.

## Results

**Table 1:** 

	LONGISSIMUS			MULTIFIDUS		
	**b_1_c**	**b_2_c**	**b_1_b_2_**	**b_1_c**	**b_2_c**	**b_1_b_2_**

**Primary**	0	100	46	0	19	10

**Secondary**	0	0	0	0	4	1

**Intermediate**	0	5	0	0	1	0

## Conclusion

Almost all lumbar muscle spindle afferents showed static sensitivity responding as primary endings terminating on b_2_ fibers. Approximately 1/3 of the afferents responded as primary endings terminating on both b_1_ and b_2_ fibers. No endings were exclusively sensitive to the dynamic ramp stimulus.

